# Improving Non-specific Immunity to Coronavirus Disease (COVID-19) by the Novelty, Diversity, and Quantity of Antigen

**DOI:** 10.3389/fpubh.2020.00393

**Published:** 2020-07-28

**Authors:** Patrice Boucher, Roger Boucher

**Affiliations:** ^1^Software and Information Technology Engineering Department, École de Technologie Supérieure, Montréal, QC, Canada; ^2^Médicentre, LaSalle, QC, Canada

**Keywords:** coronavirus, COVID-19, natural killer cells, vaccination, non-specific immunity, pandemic crisis

## The Objective: Save Lives

The health crisis caused by the COVID-19 pandemic continues to claim thousands of lives around the world. The current challenge for the scientific community, along with governments, is to quickly find solutions to save lives and limit the consequences of the crisis.

The scientific community is currently tackling the problem primarily by aiming to develop a vaccine to enable the body to develop an antigen-specific immune response to COVID-19. This approach requires time-consuming studies in order to understand the underlying properties of COVID-19 and to deploy the vaccine (1), which is very challenging to achieve at pandemic speed (2). Moreover, it could be compromised by mutations in the COVID-19 genome (3).

However, it is essential to note that the problem could be approached from at least one other front for more rapid deployment in a pandemic context. Too little consideration has been devoted to the roles of the non-specific immune response via natural killer (NK) cells, which continue to be neglected in research into vaccines (4).

## An Alternative Approach: Exploiting The Action Of NK Cells

NK cells are involved in innate resistance that is not antigen-specific, but these cells also play roles in adaptive immunity by favoring the development of antigen-specific T helper (Th) type 1 cells by producing IFN-γ and IL-2 (5–7). In other words, a better NK effector cell response [via the production of IFN-γ and the exocytosis of cytotoxic granules (4)] directly contributes to virus neutralization and to the efficiency by which specific antigens are developed at the time of infection. During an infection, NK cells also specialize as memory NK cells, which mediate protection against a second infection by the same pathogen (4, 8). The ability of these memory cells to mediate protection against other pathogens should therefore be further investigated.

Since the beginning of the pandemic, children have presented the highest resistance to COVID-19, but this resistance gradually decreases with age (9). In the first phase of infection, the innate immune system of children typically neutralizes the virus, but this is ineffective in the older population, which commonly lacks NK cells during this phase (10). The depletion of NK cells correlates with the severity of infections (10, 11). Therefore, the perspective of enhancing the response of the non-specific immune system should be seriously investigated in the context of this pandemic.

## Improving Immunity To COVID-19

Horowitz et al. (4) demonstrated that vaccination enhances NK cell effector responses in an antigen-specific manner for a fairly long period of time (their tests were conducted after about 4 months), which agrees with the documented properties of memory NK cells (8). Hence, if multiple vaccines enhance the NK effector response with diverse memory NK cells, this suggests that they may significantly contribute to (i) the neutralization of the COVID-19 pathogen, and (ii) the development of specific antibodies at the time of infection.

Children experience many novel antigens via vaccines, influenza, and other environmental pathogens. The speed by which NK cells neutralize the pathogen, as seen in children, is the main factor limiting its propagation in the body. The literature shows that the response of the immune system can be trained by episodic infections. We propose the hypothesis that the *novelty, diversity, and quantity of antigen could play a key role in training the non-specific immune system to evoke a fast, efficient response to pathogens*.

In order to test this hypothesis, we aim to reproduce the experience of children with many diverse and potentially novel antigens, before analyzing the effects of such experiences on the immune system. The complexity of this task should require numerous research projects, giving rise to a new avenue for research in immunology. Nevertheless, this could be investigated in the first instance by the revaccination of older people with a set of vaccines administered to young children. For the novelty of antigen, the use of vaccines that are new to a target population (e.g., existing vaccines in foreign countries) could also be studied. In such a study, a group of vaccinated subjects and a group of demographically equivalent non-vaccinated subjects should be periodically tested for COVID-19. If vaccination effectively improves non-specific immunity, the infected subjects of the vaccinated group should be less severely infected on average (including lower morbidity and more asymptomatic cases) than the infected subjects in the non-vaccinated group. Here, an improvement in non-specific immunity is expected, in particular, if (i) an increased concentration of NK cells is observed and lasts for several months/years; (ii) these NK cells (including primitive NK cells and memory NK cells developed in response to vaccination) are effective against new antigens; and (iii) they accelerate the development of antibodies.

It is worth mentioning that vaccines have shown to be less effective on older people, who are subject to *immunosenescence* (12). In spite of the adverse events reported, e.g., by the Vaccine Adverse Event Reporting System (VAERS) in the U.S., several vaccines have proven to be secure and relevant for adults and older people including, for example, the vaccines for measles, mumps, and rubella (13); influenza (14); pneumococcal conjugate with PCV13 and PCV23 (15); tetanus toxoid, reduced diphtheria, and acellular pertussis (16, 17); herpes zoster (18); and acute upper respiratory tract infection with the Bacillus Calmette-Guérin vaccine (19, 20), which is also under study for improving immunity to COVID-19 (21).

Yu et al. (22) analyzed the effect of childhood vaccinations on cross-reactivity against SARS-CoV in mice by evaluating the ability of T cells to recognize the SARS-CoV antigen in vaccinated compared to unvaccinated mice. They did not observe a significant difference between the two groups. Their conclusion was that “the reduced symptoms among children infected by SARS-CoV may be caused by other factors [than vaccination]”; however, this ignores the effects of the non-specific immune system via NK cells. First, as suggested by Horowitz et al. (4), vaccines given to children could be rather beneficial by improving the response of NK cells. Second, given the interspecies differences in NK cell activity (23), we do not know whether NK cells neutralize the virus prior to the development of specific antibodies.

## Conclusion

We propose the hypothesis that the *novelty, diversity, and quantity of antigen could play a key role in training the non-specific immune system for a fast, efficient response to pathogens*. If true, it is expected that vaccination with existing vaccines could help vulnerable populations to fight the virus more effectively. This hypothesis should be seriously considered since:

- It is supported by the recently documented action of the non-specific immune system via NK cells, and is coherent with clinical observations (despite the fact that they are still not sufficient to validate it). For example, this hypothesis would explain why children are more resistant to COVID-19 and why immunity gradually decreases with age.
- This could mean that thousands of lives can be saved quickly.

It could be addressed empirically by a statistical analysis of the occurrence of COVID-19 and severity in a targeted population in which we administer a set of existing vaccines. This approach could also be tested in resource-restricted settings. Most countries around the world, including developing countries, currently have access to many low-cost vaccines (24).

Given the urgency of the situation, we recommend that the scientific community, in cooperation with governments, rapidly investigate this hypothesis. Such a study would be quick, easy, and inexpensive to perform, with little risk to the population as the effects of existing vaccines are already known and validated.

## Author Contributions

RB initiated the proposal ideas. PB developed the ideas and prepared the proposal with the support of RB. All authors contributed to the article and approved the submitted version.

## Conflict of Interest

The authors declare that the research was conducted in the absence of any commercial or financial relationships that could be construed as a potential conflict of interest.
